# The Measurement of the Oxidative Index of Polyethylene Obtained during Revision Hip Arthroplasty and Assessment of Its Variability Depending on the Degree of Osteolysis, Implantation Time, as Well as the Size and Material of the Utilized Head

**DOI:** 10.3390/jcm13102751

**Published:** 2024-05-07

**Authors:** Hanna Sikora, Jadwiga Gabor, Robert Roczniok, Damian Kusz, Andrzej Swinarew

**Affiliations:** 1 Scanmed Sport Clinic, ul. Bankowa 2, 44-244 Żory, Poland; 2 Faculty of Science and Technology, University of Silesia in Katowice, ul. 75 Pułku Piechoty 1A, 41-500 Chorzów, Poland; jadwiga.gabor@us.edu.pl; 3 Department of Sport Theory and Practice, The Jerzy Kukuczka Academy of Physical Education in Katowice, ul. Mikołowska 72A, 40-065 Katowice, Poland; r.roczniok@awf.katowice.pl; 4 Department of Orthopedics and Traumatology, Medical University of Silesia, ul. Ziołowa 45/47, 40-635 Katowice, Poland; damian.kusz@sum.edu.pl; 5 Department of Swimming and Water Rescue, Institute of Sport Science, The Jerzy Kukuczka Academy of Physical Education, ul. Mikołowska 72A, 40-065 Katowice, Poland

**Keywords:** polyethylene wear, osteolysis, total hip replacement, polyethylene failure, revision total hip replacement

## Abstract

**Background/Objectives:** Aseptic loosening is the leading cause of late revision in total hip arthroplasty, primarily due to degenerative oxidation of polyethylene components, leading to wear particle formation and periacetabular osteolysis. This study aimed to analyze the oxidation levels in polyethylene liners and cemented cups retrieved from revision surgeries using Fourier-transform infrared spectroscopy (FTIR) and to explore the correlation between oxidation levels and factors such as head size, head material, fixation method, and implant survival time. **Methods:** Polyethylene liners and cups were analyzed post-revision surgery to assess oxidation levels, which were then compared to periacetabular bone loss measured by the Paprosky classification. This study evaluated the impact of head size (28 mm vs. 32 mm), head material (ceramic vs. metal), and fixation methods on oxidation. The relationship between the mean oxidation index (OI) and implant survival time was also investigated. **Results:** There was a significant positive correlation between the mean oxidation index of the polyethylene components and the severity of periacetabular osteolysis according to the Paprosky scale. While the mean OI for samples articulating with ceramic heads was lower than for those with metal heads, and the mean OI for samples with a 32 mm head size was lower than for those with a 28 mm size, these differences were not statistically significant. Furthermore, the fixation method did not affect the oxidation index, and no correlation was found between OI and the survival time of the implants. **Conclusions:** This study confirms a direct correlation between polyethylene oxidation and periacetabular osteolysis in hip replacements, highlighting the importance of material choice and design in potentially reducing the risk of aseptic loosening. Despite the lack of significant differences in oxidation levels based on head material and size, these factors may still play a role in the long-term outcome of hip arthroplasty, warranting further investigation.

## 1. Introduction

Polyethylene (PE), encompassing ultra-high molecular weight PE (UHMWPE) (also referred to as ‘standard’ or ‘conventional’ PE) and crosslinked UHMWPE (XLPE), remains a common choice as a bearing surface in total hip arthroplasty [1]. It is utilized either as a cemented all-polyethylene cup or as a liner integrated into the metal cup. The prevalent configuration of hip implants typically involves a metal alloy femoral stem, a head crafted from ceramics or metal alloys (commonly cobalt–chromium), articulating against the polyethylene surface (acetabular cup/liner). The utilization of alternative bearing materials, such as metal-on-metal (MoM) or ceramic-on-ceramic (CoC), raises concerns regarding costs, biocompatibility, and revision difficulties. This renders metal-on-XLPE or ceramic-on-XLPE the gold standard in hip arthroplasty [1]. According to the Swedish Arthroplasty Register, in 2021, XLPE was employed in 99% of all cases of uncemented total hip arthroplasty [2].

Introduced in 1962, ultra-high molecular weight polyethylene (UHMWPE) was the material of choice for bearing surfaces in total hip replacements until the last two decades, when it was gradually replaced by XLPE [1,3]. It has been reported that particles produced by the wear of UHMWPE liners articulating with metallic or ceramic heads have caused peri-prosthetic osteolysis, leading to loosening and instability of implants [3,4]. One of the main factors responsible for the late failure of UHMWPE in hip replacement is degenerative oxidation, resulting in the formation of wear particles [4]. To prevent oxidative degradation and polyethylene wear, highly crosslinked UHMWPE (XLPE) was developed [3]. Highly crosslinked UHMWPE was clinically introduced in the 1990s and has become the gold standard in the last two decades [1]. XLPE is described as UHMWPE that has been irradiated with at least 50 kGy of gamma (or beta) or electron beam radiation [1,3]. This process significantly increases the number of crosslinks between polyethylene chains, thereby improving its wear resistance [1,3]. Although it has been reported that XLPE exhibits decreased wear and osteolysis compared to UHMWPE [5], gamma radiation in the air not only causes increased polyethylene crosslinking but also results in free radicals, which are susceptible to oxidation. Therefore, it can escalate osteolysis and wear [6]. It has been shown that gamma irradiation with barrier packaging without oxygen results in a lower potential for oxidation [6,7]. However, there is evidence that in vivo oxidation of residual free radicals remains a possibility in those inlays [7]. Apart from irradiation, the melting technique and annealing can also affect the in vivo properties of XLPE and are used to improve their oxidation resistance by quenching the free radicals [8]. XLPE liners that undergo annealing after irradiation usually result in the retention of some oxidation potential but show good wear resistance [1,8]. On the contrary, XLPE liners that undergo remelting after irradiation show good oxidation resistance but less fatigue resistance [1,8]. To achieve oxidation resistance after the radiation of crosslinked UHMWPEs, the diffusion of the antioxidant vitamin E was developed [9]. E1-polyethylene does not wear more than conventional PE [9,10]. Despite these polyethylene improvements, aseptic loosening is still the most common reason for late revision in total hip arthroplasty (THA) [1]. According to the Swedish Arthroplasty Register 2022, it is responsible for 42.2% of revision surgeries [2].

In light of the ongoing challenges with polyethylene implants in total hip arthroplasty, this study aims to further investigate the in vivo performance and long-term outcomes of XLPE liners. The primary objective of this study was to establish the durability of total hip arthroplasty and to address the influence of the oxidation process on overall failure rates. The secondary objectives include evaluating the biomechanical stability and patient outcomes associated with different implant types over a long-term period.

## 2. Materials and Methods

A total of 51 patients who underwent revision surgery for total hip arthroplasty at the Department of Orthopedics and Traumatology at the Medical University of Silesia from 2018 to 2021 were included in our study. The objective of this experiment was to employ Fourier-transform infrared spectroscopy (FTIR) for the analysis of polyethylene liners and cemented polyethylene cups retrieved during revision surgeries. The primary focus was on assessing their oxidation levels, comparing them to periacetabular bone loss measured according to the Paprosky classification. Additionally, this study aimed to evaluate oxidation levels in relation to head size, head material, fixation method, and survival time. Eighteen patients were excluded from the analysis due to meeting exclusion criteria (septic loosening, traumatic loosening, revision surgery due to dislocation of the prosthesis).

All liners and cups were mechanically cleaned and soaked in disinfectant. Following this, they were promptly placed in sterile barrier packaging to prevent exposure to oxygen. Samples were then obtained using a sterile scalpel from both the backside and bearing surface (the most worn superior surface) of a cup or inlay. FTIR spectra of both sides of the samples were measured. As a control sample for our study, we used a new, sterile, unused inlay made of highly crosslinked UHMWPE, which was provided by one of the orthopedic manufacturing companies for the purpose of this study.

The FTIR analyses were conducted using the Shimadzu IR Prestige-21 Fourier Spectrometer. Spectra were obtained in transmittance mode within the spectral region of 550–4000 cm^−1^. To identify the functional groups of the samples, the ATR FTIR technique was employed. This spectroscopic method is recognized for its accuracy, enabling the assignment of specific functional groups to distinct areas through the analysis of characteristic absorption bands.

By measuring the intensity of light across a spectrum of infrared wavelengths post-interaction with the sample, FTIR can effectively identify specific molecular vibrations. These vibrations correspond to various chemical bonds and molecular structures, thus generating a unique spectral fingerprint of the material. This capability is especially crucial in identifying functional groups such as carbonyl groups, which are indicative of oxidative changes in materials. The selection of FTIR for this study was driven by its high sensitivity and specificity to oxidative degradation processes in polyethylene components used in total hip arthroplasty. Oxidation is a significant factor that can lead to material degradation and is a potential precursor to implant failure. By quantifying the extent of oxidation through an oxidation index derived from FTIR spectra, this study aims to correlate material degradation with clinical outcomes such as the longevity and failure rates of the implants. FTIR additionally offers the advantage of being non-destructive, allowing for the preservation of samples for further analysis. This attribute is vital in medical research where sample integrity is paramount.

By integrating FTIR into our analytical approach, we aim to provide a comprehensive evaluation of the oxidative state of polyethylene liners and cups, facilitating a better understanding of their performance and durability in clinical settings.

In accordance with ASTM F2102-17, the degree of oxidation was quantified using an oxidation index. This index, calculated from FTIR spectra, represents the area ratio between two specific regions, 1685–1745 cm^−1^, indicative of carbonyl group absorbance and ester formation, and 1330–1390 cm^−1^, serving as a reference absorbance to normalize for the sample’s thickness and account for methylene stretching [11].

OI = 1685–1745 cm^−1^/1330–1390 cm^−1^

Calculated values were compared according to the survival time of the implant, head type (metal/ceramics), head size, periacetabular osteolysis, and fixation method (cement/cementless). Periacetabular osteolysis was classified into 6 types (1, 2A, 2B, 2C, 3A, and 3B) according to Paprosky classification by two surgeons. The categorization was based on preoperative X-ray analysis.

The Paprosky classification system was devised to anticipate the need for augmentations during revision surgery. It categorizes patterns based on the degree of superior migration of the hip center compared to the obturator line, as well as the integrity of key anatomical landmarks such as the teardrop, ischium, and Koehler’s line observed on preoperative pelvic AP radiographs. Acetabular columns are then classified into three types: completely supportive (type I), partially supportive (type II), and non-supportive (part III). This classification system continues to be extensively utilized and has shown robust inter- and intra-observer reliability. Another commonly employed classification system is the AAOS classification. It is based on intraoperative assessment and delineates osteolysis by pattern (segmental/cavitary/combined/pelvic discontinuity or arthrodesis), further subclassified by location (superior, anterior, posterior, or central). For scientific research purposes, the Paprosky classification is more accessible—it is systematic and easier to apply in clinical practice compared to descriptive classification; the degree of osteolysis is established based on a single X-ray image. This classification is also favored by surgeons because it dictates the approach to defect management during revision surgery, depending on the degree of osteolysis.

Statistical analysis was performed using the U Mann–Whitney test and Kruskal–Wallis test, with a significance level of *p* < 0.05. The association between variables was measured using Spearman’s correlation. This study obtained approval from the Ethics Committee of the Medical University of Silesia.

## 3. Results

We examined 33 polyethylene samples, comprising 11 cemented cups and 22 poly-ethylene inlays (from 13 treated cups and 9 press-fit cups) revised due to aseptic loosening. Of these, 21 polyethylene samples were articulating with metal head sizes 28 mm (17 samples) and 32 mm (4 samples). The remaining 12 samples were articulating with ceramic head sizes: 28 mm (8 samples) and 32 mm (4 samples). The 28 mm head size accounted for 75.76% (25/33), while the 32 mm head size represented 24.24% (8/33). Metal heads constituted 63.64% (21/33), and ceramic heads accounted for 36.36% (12/33) of the revised implants included in this study.

The index procedure was performed between 1993 and 2020. The mean survival time of the implants was 14.42 years (SD = 6.67), ranging from a minimum survival period of 1 year to a maximum of 28 years. No correlation was found between the oxidation index (OI) and the survival time of the bearing surface of the samples (R = 0.25; *p* = 0.16) or the backside of the samples (R = 0.22; *p* = 0.23). Additionally, there was no statistically significant difference in mean survival time between the metal and ceramic head groups (14.2857 vs. 14.6666).

We observed a higher mean oxidation index of the bearing surface of the inlays (1.60, on average; range: 0.45–3.91) compared to the backside (1.48, on average; range: 0.49–4.08), but the difference was not statistically significant (*p* = 0.10). However, a strong correlation was observed between the degradation of the bearing surface and the backside of the inlays—increased OI of the bearing surface was strongly associated with increased OI of the backside (*p* = 0.0005). The mean oxidation index of the bearing surface for samples articulating with a ceramic head was lower than for those articulating with a metal head (1.55 vs. 1.63); however, the difference was not statistically significant (*p* = 0.96). Similarly, there was no statistically significant difference (*p* = 0.44) between the mean oxidation indices of the bearing surface for samples articulating with a 28 mm head size compared to a 32 mm head size (1.64 vs. 1.48).

The most commonly revised cup, based on fixation type, was the cementless threaded cup (13 out of 33 implants, accounting for 39.39%). Cemented cups represented 11 out of 33 implants (33.33%). The least frequently revised implants were press-fit cups (9 out of 11 implants, accounting for 27.27%). Our study showed no statistically significant differences in oxidation indices according to the type of cup fixation—cemented vs. press-fit vs. threaded (1.43 vs. 1.50 vs. 1.82, respectively, for the bearing surface, *p* = 0.31; and 1.53 vs. 1.60 vs. 1.36, respectively, for the backside, *p* = 0.85). The aforementioned descriptive statistics are presented in Table 1.

The oxidation index of the control sample was immeasurable—there were no measurable peaks in the carbonyl group spectrum (1685–1745 cm^−1^).

**Figure 1:**
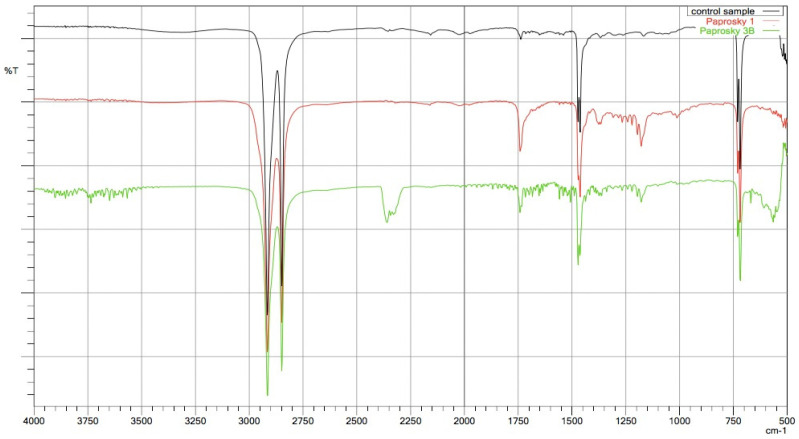
FTIR spectra differences in oxidative indices between the control sample and selected samples from groups 1 and 3B.

**Figure 2:**
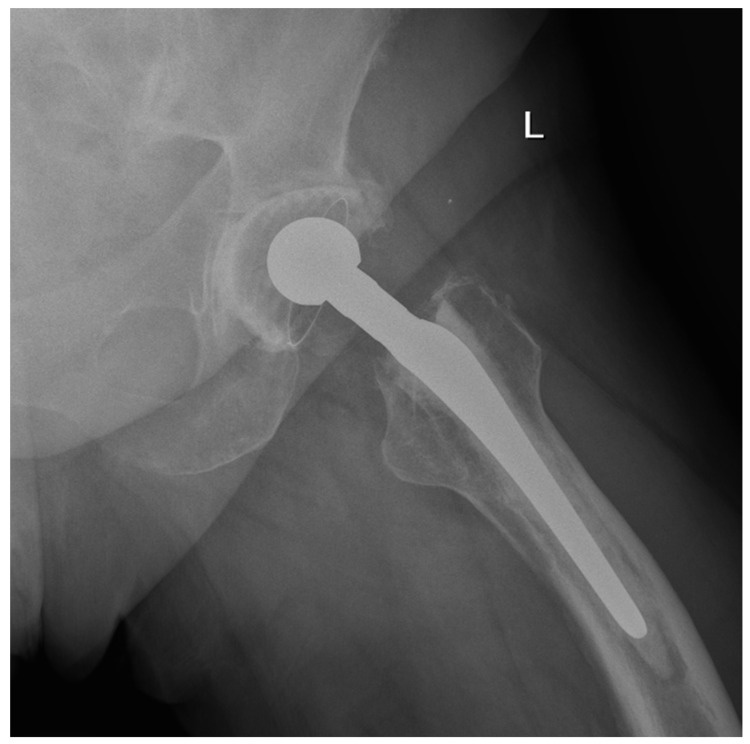
Periacetabular osteolysis classified as Paprosky 1.

**Figure 3:**
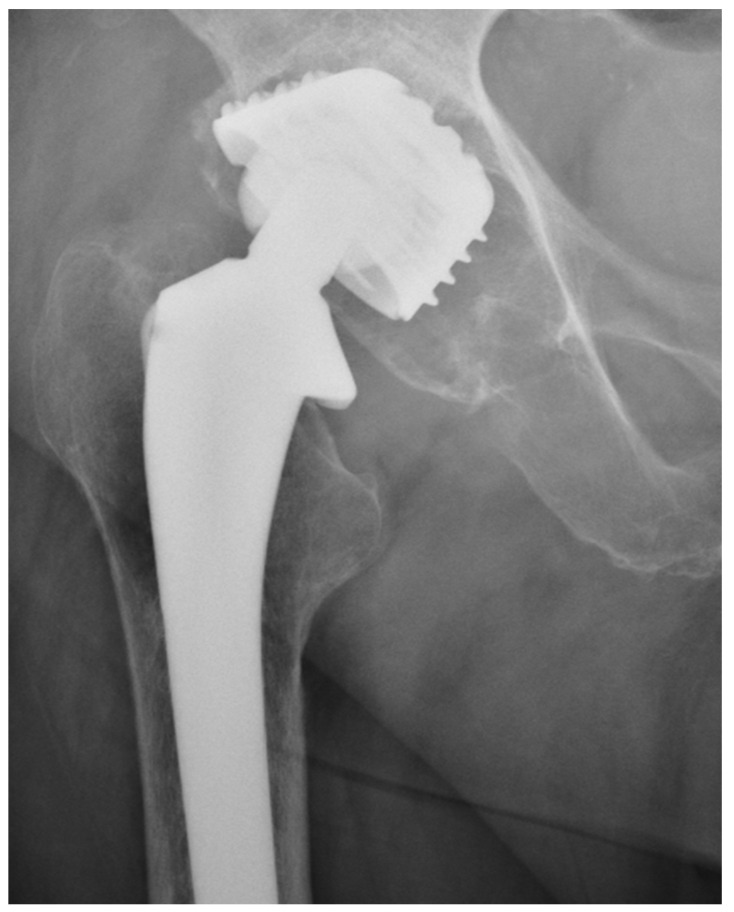
Periacetabular osteolysis classified as Paprosky 2B.

**Figure 4:**
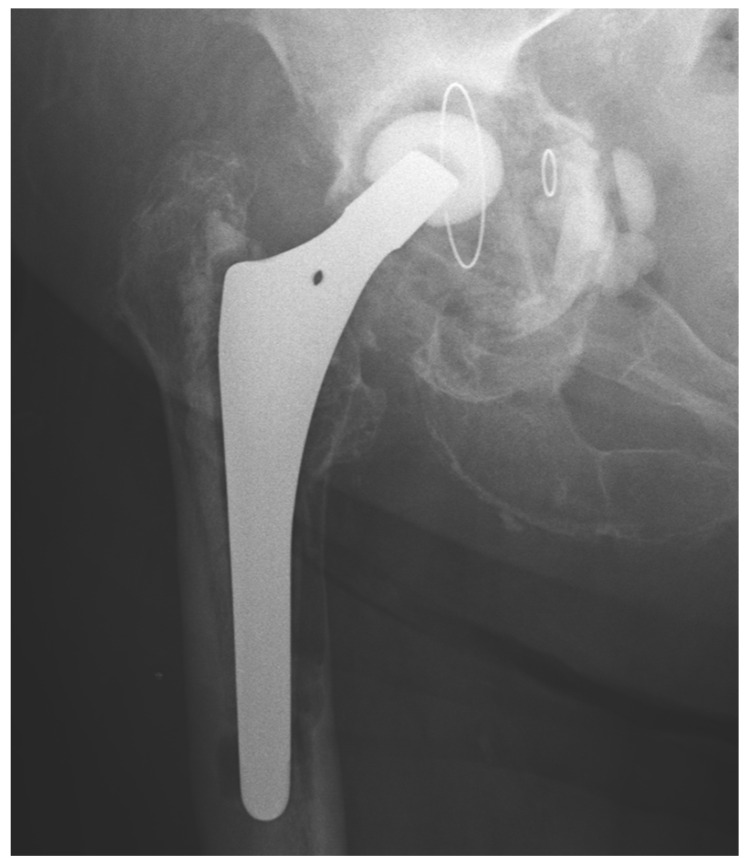
Periacetabular osteolysis classified as Paprosky 3B.

We observed a positive correlation between the increase in the mean oxidation index (OI) of the bearing surface of the inlays and the extent of periacetabular osteolysis classified according to the Paprosky scale (R = 0.44; *p* = 0.011). The most commonly revised types were 3A and 2B: 12 out of 33 samples (36.36%) were classified as type 3A, and 7 out of 33 samples were classified as type 2B (21.21%) according to Table 2. Mean oxidation index values for each type are, respectively, presented in Table 3. Figure 1 illustrates the differences in oxidative indices between the control sample and selected samples from groups 1 and 3B. Attention should be paid to the difference in the spectrum range of 1685–1745 cm^−1^. Representative X-ray images of patients depicting the extent of osteolysis based on the Paprosky scale can be seen in Figure 2, Figure 3 and Figure 4.

## 4. Discussion

According to the literature, the extent of oxidative degradation appears to be a key parameter; the higher the oxidation index, the poorer the mechanical properties and wear resistance [12,13]. The close correlation between oxidative degradation and the performance of UHMWPE liners is discussed in the *UHMWPE Biomaterials Handbook* by Kurtz and Oral [12]. As stated by the authors, low oxidation (OI < 1) implies no adverse impact on mechanical properties, while critical oxidation (OI > 3) indicates a reduced ability of the material to withstand long-term mechanical loading in vivo [12]. The outcomes of our study align with the aforementioned findings: the mean oxidation index of the bearing surface of the retrieved inlays was 1.60, and only 3 out of 33 samples had an oxidation index below 1. The backside of the inlay exhibited a lower degree of degeneration, with a mean oxidation index of 1.48, and 8 out of 33 samples had an oxidation index below 1.

The proven deterioration of mechanical properties does not necessarily have a negative impact on the clinical performance of the liners—our study did not show a correlation between the survival time and the oxidation index. A similar study by Kurtz et al. [14] demonstrated that rim oxidation significantly increased with implantation time; nevertheless, a positive trend was not detected at the bearing surface and backside [14]. It is noteworthy that the inlays were revised after an average of only 2.9 years (range: 0.01–8.0 years) for reasons unrelated to wear or mechanical performance.

The study conducted by Choudhury and colleagues also suggested that there was no correlation between the duration of implantation and the measured oxidation index [13]. They also established that the levels of OI were predominantly elevated in the wear and rim regions and were mostly correlated with wear depth [13].

Numerous studies have suggested that polyethylene wear and oxidation lead to the production of particles, which, in turn, can cause periacetabular osteolysis [1,4,15,16]. However, there is limited literature comparing the oxidation index with osteolysis. Schachtner et al. [17] compared wear, oxidation indices, and osteolysis outcomes between annealed and remelted first-generation highly crosslinked polyethylene acetabular cup liners. While the annealed cohort showed elevated oxidative indices (OIs) in comparison to the remelted cohort, there were no discernible differences in the reported incidence of osteolysis between the two groups [17]. The review by Kurz et al. [18] demonstrated an 87% lower risk of osteolysis with HXLPE liners. Consistent with our findings, the reduction in femoral head penetration or osteolysis risk remains unestablished for large-diameter (>32 mm) metallic femoral heads or ceramic femoral heads of any size [18].

The choice of fixation method for the acetabular component of the prosthesis depends on the quality of the patient’s bone tissue—in older patients and those with osteoporosis, the gold standard remains a cemented acetabular component. In younger patients with good bone quality, the standard remains cementless acetabular components, commonly implanted as press-fit cups. Threaded cups, once popular, are now not widely used, despite some studies showing no advantage of press-fit cups over threaded cups in terms of implant survival and functional results [19,20,21]. Surgeons’ main concern is increased osteolysis around the cup and the difficulty in removing the cup during revision, which significantly complicates potential implant replacement. Our study revealed no statistically significant differences in polyethylene oxidation regardless of the fixation method. Scientific research shows that the fixation method does not significantly impact implant survival, long-term functional outcomes, or polyethylene wear [22,23].

Despite numerous conclusions, our study has three main limitations. The first one is the small number of samples; however, the findings of this study offer new, potentially useful information for this patient population. The second limitation is that the degree of crosslinking and the type of thermal treatment (remelting vs. annealing) in the analyzed polyethylene remain unknown. The primary goal of this study was to establish the durability of total hip arthroplasty (THA) from 1993 to 2020 and to address the influence of the oxidation process on general failure rates. Finally, we did not control for patients’ factors, such as activity level, gait pattern, metabolic diseases, BMI, sex, and age. However, a review of the literature found no statistically significant association between the amount of wear per year and patient age, weight, and sex [13,19].

In scientific studies, there are nevertheless reports that the level of activity and gait pattern affect the wear of polyethylene [24,25]. In this study, however, it was impossible to determine the gait pattern exhibited by patients from the time of primary total hip arthroplasty to the time of revision. Patients were physically examined before admission to the hospital for hip prosthesis replacement due to loosening. At that time, the majority of them presented with a limping gait, as the main symptom of implant failure is hip pain. Also, the level of activity was impossible to measure; discussing the activity level with the patient from the time of the index procedure to admission to the hospital would have been solely a subjective assessment.

## 5. Conclusions

In summary, oxidative degradation of polyethylene inlays appears to be correlated with osteolysis; however, it does not affect the long-term clinical performance of the implants. In comparison with literature reports, the degeneration and wear of polyethylene in hip arthroplasty, as well as their correlation with osteolysis, seem to be a more complex, multifactorial issue, requiring further research.

Although oxidative degradation of polyethylene liners has been correlated with osteolysis, our findings also highlight that, despite variations in oxidation levels, there was no significant impact on the overall survival time of the implants. This highlights the need for further investigation into the direct effects of polyethylene oxidation on other clinical performance indicators beyond implant survival, to fully understand its role in the longevity and efficacy of hip arthroplasty.

## Figures and Tables

**Table 1:** Descriptive statistics, normality of distributions, and tests of significance of differences for the analyzed variables.

Group	Variable	M	CI −95%	CI 95%	Me	Min	Max	SD	*p* S-W	Z/H	*p*
Metal N = 21	OI—bearing surface	1.63	1.29	1.97	1.48	0.45	3.91	0.75	0.065	0.06	0.96
Ceramics N = 12		1.55	1.31	1.79	1.58	1.08	2.35	0.37	0.35		
Metal N = 21	OI—backside	1.45	1.10	1.80	1.24	0.63	4.08	0.77	<0.0001	−0.32	0.75
Ceramics N = 12		1.54	0.97	2.12	1.43	0.49	4.01	0.91	0.020		
Head size 28 N = 25	OI—bearing surface	1.64	1.36	1.93	1.51	0.45	3.91	0.69	0.025	0.78	0.44
Head size 32 N = 8		1.48	1.12	1.84	1.49	1.03	2.35	0.43	0.18		
Head size 28 N = 25	OI—backside	1.49	1.18	1.79	1.27	0.63	4.08	0.73	0.0002	0.65	0.51
Head size 32 N = 8		1.48	0.57	2.38	1.21	0.49	4.01	1.09	0.008		
Cementless threaded cup N = 13	OI—bearing surface	1.82	1.34	2.30	1.74	0.78	3.91	0.79	0.062	H (2. N = 33) = 2.32	0.31
Cemented cup N = 11		1.43	1.12	1.74	1.34	0.76	2.39	0.46	0.62		
Press-fit cups N = 9		1.50	1.10	1.90	1.50	0.45	2.35	0.52	0.48		
Cementless threaded cup N = 13	OI—backside	1.36	1.10	1.62	1.23	0.75	2.31	0.42	0.25	H (2. N = 33) = 0.32	0.85
Cemented cup N = 11		1.53	0.86	2.20	1.24	0.81	4.08	1.00	0.001		
Press-fit cups N = 9		1.60	0.80	2.40	1.48	0.49	4.01	1.04	0.04		
Total N = 33	OI—bearing surface	1.60	1.38	1.83	1.50	0.45	3.91	0.63	0.007	1.64	0.10
	OI—backside	1.48	1.20	1.77	1.27	0.49	4.08	0.81	<0.0001		

M—mean; CI95%—95% confidence interval; Me—median; Min—minimum; Max—maximum; SD—standard deviation; *p* S-W—test probability for the test of normality of distribution; Z/H—Z or H test results; *p*—test probability.

**Table 2:** Frequency and percentage characteristics for the Paprosky scale.

Type	Paprosky Scale	
	Number of Samples	Percent
‘1’	3	9.09
2A	3	9.09
2B	7	21.21
2C	4	12.12
3A	12	36.36
3B	4	12.12

The table presents a quantitative distribution of samples based on the Paprosky classification grades and their percentage distribution.

**Table 3:** Average values for individual classes of the Paprosky scale.

Paprosky Scale (Type)	Mean OI—Bearing Surface
‘1’	1.178
2A	1.144
2B	1.495
2C	1.538
3A	1.677
3B	2.300

The table presents the average oxidative index for each Paprosky classification grade.

## Data Availability

To share data, please contact us via email (hanna.sikora@outlook.com).
